# Editorial: Neuro-immune interplay: unraveling the complexities of neurological complications and immunology

**DOI:** 10.3389/fnmol.2025.1607675

**Published:** 2025-04-30

**Authors:** Pankaj Gaur, Kumar Vaibhav, Meenakshi Ahluwalia, Seema Gupta

**Affiliations:** ^1^Lombardi Comprehensive Cancer Center, Georgetown University Medical Center, Washington, DC, United States; ^2^Department of Neurosurgery, Medical College of Georgia, Augusta University, Augusta, GA, United States; ^3^Department of Pathology, Medical College of Georgia, Augusta University, Augusta, GA, United States; ^4^Georgia Cancer Center, Medical College of Georgia, Augusta University, Augusta, GA, United States

**Keywords:** neuroinflammation, cytokine signaling, neurological complications and immunology, T cell, astrocytes and microglia

A growing body of evidence suggests that immune-mediated mechanisms significantly contribute to the pathogenesis of autoimmune diseases (AID), neurodegenerative conditions, and pain disorders (Shastri et al., 2023; Jain et al., 2024; Mason and McGavern, 2022). Indeed, a complex interplay between immune dysregulation, neuroinflammation, and neurological dysfunction has emerged as a critical focus in biomedical research. In this editorial, we synthesize findings from six recent studies that examine diverse yet interconnected aspects of neuro-immune interactions, spanning AID, paraneoplastic neurological syndromes (n-PNS), disc herniation (DH), intracerebral hemorrhage (ICH), Alzheimer's disease (AD), and trigeminal neuralgia (TN). These studies provide novel insights into inflammatory pathways, cytokine dynamics, and potential therapeutic targets that may shape future clinical approaches.

The original study by Cox et al. explored the impact of systemic inflammation from rheumatoid arthritis (RA) and ulcerative colitis (UC) on the brain. Using neuroimaging data from the UK Biobank, the researchers identified significant volumetric differences: patients with UC exhibited significant hippocampal atrophy, while RA was associated with reduced amygdala volume. Hippocampal atrophy has been implicated in cognitive decline and mood disorders (Opel et al., 2014; Belleau et al., 2019; Rao et al., 2022). Similarly, amygdala dysfunction is often linked to increased susceptibility to anxiety and depression (Hu et al., 2022; Grogans et al., 2022; Klug et al., 2024). Collectively hippocampus and amygdala constitute the interconnected components of the limbic system that control emotions, memory, arousal and motivational behaviors (Rajmohan and Mohandas, 2007; Voss et al., 2017; Immordino-Yang and Singh, 2013). Thus, any alterations in the morphology or function of these components affect limbic system significantly (Yang and Wang, 2017). These observations suggest that AID not only affect peripheral organs but also have profound implications on the central nervous system, and thus, reinforcing the need for integrated neuropsychiatric care in patients with AID.

Paraneoplastic syndromes (PNS) represent an enigmatic intersection between cancer and autoimmunity and are associated with a range of affected systems, including neurologic, endocrine, dermatologic, and others (Gilligan et al., 2023; Soomro et al., 2020). n-PNS is characterized by neurological symptoms and majority of times is associated with lymphomas, and lung, breast, and ovarian cancers (Graus et al., 2021; Gilligan et al., 2023). In a retrospective study, Bar Mucha et al. examined several factors, including long-term survival and quality of life in patients with positive onconeural antibodies, who developed cancer, and exhibited a recognizable PNS phenotype. The study revealed that while there was a no difference in mortality among patients who initially presented with n-PNS or with cancer, these disorders significantly impacted patient's quality of life as there was an increase in long term disability and functional impairment. Although this study was conducted in a small cohort of 12 patients, the findings suggest that further research and multidisciplinary collaborations are essential for improving treatment outcomes and developing effective approaches for patients with PNS (Bar Mucha et al.).

Chronic pain conditions such as DH and TN are often associated with prolonged stress, activated immune cells, and elevated cytokines which manifest as anxiety, mood disorder and depression (Bielewicz et al.; Kao et al., 2021; Kayhan et al., 2016; Alwardian et al., 2021; Cheng et al.), yet the underlying mechanisms remain poorly understood. The study by Bielewicz et al. demonstrated elevated serum levels of both pro-inflammatory (IL-6) and anti-inflammatory (TGF-β and IL-10) cytokines in symptomatic DH patients with moderate to severe depressive (SD) symptoms compared to patients with mild (MD) or no depressive (ND) symptoms. Despite increased serum levels of TGF-β and IL-10, which are involved in healing processes, the functional impairment was more severe in patients with SD symptoms. This paradoxical increase in anti-inflammatory cytokines suggests a compensatory response to ongoing neuroinflammation, yet it remains insufficient in mitigating depressive symptoms. This supports the “sickness behavior” hypothesis, where systemic inflammation drives behavioral changes akin to major depression. These findings emphasize the importance of a multidisciplinary approach in treating chronic pain, incorporating both pain management and mood disorder interventions.

In another novel study, Cheng et al. explored the role of Stromal Interaction Molecule 1 (STIM1) in inflammatory cytokine release in T cells in TN. TN is a debilitating neuropathic pain disorder characterized by severe, paroxysmal facial pain (Alwardian et al., 2021). Their analysis revealed that aberrant calcium signaling in T lymphocytes contributes to inflammatory cytokine release, exacerbating pain. Findings from the *in vitro* and *in vivo* experiments demonstrated that STIM1 regulates store-operated calcium entry (SOCE) pathway mediating the release of inflammatory cytokines in T cells. Inhibiting STIM1 activity reduced the inflammatory marker expression (TNF-α, IL-1β, IL-6) and alleviated pain symptoms. These findings suggest that calcium signaling in immune cells plays a pivotal role in TN pathogenesis and may serve as a novel therapeutic target. Given the limitations of current TN treatments, which include surgical interventions and pharmacologic agents with significant side effects, targeting SOCE may offer a more precise and less invasive approach.

In addition to infiltrated peripheral immune cells, residential glial cells (astrocytes and microglia) provide immune response on bases of pathological, or injury clues present in the brain (Jarrahi et al., 2020; Vaibhav et al., 2024; Linnerbauer et al., 2020; Giovannoni and Quintana, 2020). A review article by Dong et al. on ICH focused on the role of astrocytes in secondary brain injury. Astrocytes modulate blood-brain barrier, regulate oxidative stress, and influence neuronal survival, but prolonged activation can exacerbate neuroinflammation (Endo et al., 2022; Sofroniew and Vinters, 2010). The review identifies several key pathways involved in regulation of astrocyte function and reactivity, including JAK/STAT3, NF-κB, and MAPK pathways (Dong et al.; Linnerbauer et al., 2020; Giovannoni and Quintana, 2020). Multiple astrocytic proteins such as S100B, NLRP6, Prdx1, TLR2, and CK2 are involved in regulating the inflammatory response and subsequent effect on the oxidative stress, apoptosis and activation of various types of cells. Further, the mechanisms and pathways involved in the interplay of astrocytes with other cells such as, microglia and neurons following ICH, include IL-15, AQP2, TIM-3, TRPA1, HN, Homer1, lactate, galectin-9, and mitochondrial transfer. The conclusions of the studies suggest that targeting AQP4, hepcidin, and NDRG2 may be effective in mitigating cerebral edema and neurological deficits after ICH, while compounds like curcumin, carvacrol, and GHK may have therapeutic potential.

Microglia as residential immune cells clear amyloid plaque at an early stage of AD, but also contribute to exaggerated inflammation via stimulation through circulating cytokines (O'Connor and Nissen, 2023; Ocañas et al., 2023; Poppell et al., 2023). Zuppe and Reed's perspective sheds light on the role of the common cytokine receptor gamma chain family cytokines in activation of the three major signaling pathways in microglia- MAPK, PI3K, and JAK/STAT pathways and their effect in AD. While all three families of MAP kinases, ERK, JNK, and p38/SAPK stimulate inflammatory cytokine production in microglia, authors explain that only JNK and p38 may be involved in neuronal damage. The JAK/STAT pathway is responsible for quick signaling associated with pro-inflammatory responses (Millot et al., 2020). Over-activation of the JAK/STAT pathway in AD is typically associated with STAT3, likely due to STAT3 phosphorylation being increased in the hippocampus of both mouse models and postmortem brains (Millot et al., 2020). The later events might be attributed to suppressed negative regulation by dysregulated PI3K pathway-related genes (Chu et al., 2021). Thus, these three pathways have attracted quite attention for therapeutic targeting in AD treatment (Zuppe and Reed). However, only rapamycin is currently being tested in a clinical trial (NCT04200911) and a dual inhibitor of mTORC and PI3K, NVPBEZ235 has shown some therapeutic potential in an AD mouse model (Bellozi et al., 2019) and in Aβ pathology (Bellozi et al., 2016). Together, this perspective proposes that targeting common cytokine receptor gamma chain family of cytokines signaling could modulate microglial activation, reduce neuroinflammation, and potentially slow cognitive decline. These insights provide a strong rationale for exploring immunomodulatory strategies in AD therapy.

These studies collectively emphasize the significant role of immune signaling in neurological disorders. Several key themes emerge: (1) Cytokine modulation may provide novel therapeutic avenues for autoimmune, neurodegenerative, and pain-related disorders; (2) Chronic inflammation contributes to both neurological and psychiatric symptoms, necessitating an integrated approach to treatment; (3) Glial cells, particularly astrocytes and microglia, play a central role in disease progression and could serve as therapeutic targets; (4) Calcium signaling pathways, such as STIM1 in TN, represent new frontiers for drug development. The convergence of neuroimmunology and neurology is redefining our understanding of disease mechanisms and treatment strategies. As research advances, interdisciplinary collaborations between neuroscientists, immunologists, and clinicians will be crucial in translating these findings into effective therapies. Addressing neuro-immune dysfunction holds the promise of not only mitigating neurological symptoms but also improving overall treatment outcomes in a wide array of conditions.
